# Supplementary material for: Clustering ICU patients with sepsis based on the patterns of their circulating biomarkers: A secondary analysis of the CAPTAIN prospective multicenter cohort study
Source: PLoS One. 2022 Oct 27;17(10):e0267517. doi: 10.1371/journal.pone.0267517 (PMC9612564; doi:10.1371/journal.pone.0267517)
Supplement: S1 File — Techniques of biomarkers measurements. Table A. STROBE—Checklist of items that should be included in reports of cohort studies. Table B. Characteristics of the patients that were discharged or died before day3 (n = 33). Table C. Circulating biomarkers with a loading > 0.40 or < -0.40 in each main independent patterns obtained after principal component analysis. Table D. Value of each pattern of biomarkers in the identified clusters (med [Q1-Q3]). (PDF) [file pone.0267517.s001.pdf]

S1 File

Supplement to: Misset B, Philippart F, Fitting C, et al. Clustering ICU patients with sepsis based on the patterns of their circulating biomarkers: a secondary analysis of the CAPTAIN prospective multicenter cohort study. PlosOne 2022

This supplement has been provided by the authors to give readers additional information about the work

Summary

Summary......1

Methods: techniques of biomarkers measurements......2

Table A: STROBE—Checklist of items that should be included in reports of cohort studies......4

Table B: Characteristics of the patients that were discharged or died before day3 (n=33)......5

Table C: Circulating biomarkers with a loading > 0.40 or < -0.40 in each main independent patterns obtained after principal component analysis......6

Table D: Value of each pattern of biomarkers in the identified clusters (med [Q1-Q3])......7

References......8

Methods: techniques of biomarkers measurements

We collected whole blood samples at day 0 and 1 of inclusion to assess 38 biomarkers, reported as potential indicators of infection or mortality during sepsis. They were measured using various techniques: ELISA for galectin, peptidoglycan, visfatin (previously known as pre-B cell colony-enhancing factor (PBEF), soluble B7-H6 (sB7-H6), soluble Triggering Receptor Expressed on Myeloid cells-1 (sTREM-1), soluble urokinase-type Plasminogen Activator Receptor (suPAR) and Pancreatic Stone Protein (PSP); Bioplex technique for C-Reactive Protein (CRP), ferritin, Granulocyte-Colony Stimulating Factor (G-CSF), chemokines: GRO alpha (GRO-α/CXCL1), Monocyte Chemo-attractant Protein 1 (MCP-1/CCL2), Macrophage Inflammatory Protein-1 alpha (MIP-1α/CCL3), Macrophage Inflammatory Protein-1 beta (MIP-1β/CCL4), Regulated upon Activation Normal T-cell Expressed and Secreted (RANTES/CCL5), IFN-γ-induced protein 10 (IP-10/CXCL10); Interferon gamma (IFN-γ), Interleukin-1 Receptor antagonist (IL-1Ra), Interleukins 6, 8, 10, 15, and 18 (IL-6, IL-8, IL-10, IL-15 and IL-18), Macrophage-Colony Stimulating Factor (M-CSF), Macrophage Migration Inhibitory Factor (MIF), Matrix Metallo Proteinase-8 (MMP-8), Procalcitonin (PCT), and Tumor Necrosis Factor (TNF); and Real Time quantitative Polymerase Chain Reaction (RT-qPCR) for the following whole blood mRNAs: Cluster of Differentiation 3δ and 74 (CD3D and CD74), CX3 chemokine receptor 1 (CX3CR1), Human Leucocyte Antigen DR-alpha chain (HLA-DR), High Mobility Group Box 1 protein (HMGB1), IL-1β, IL-10, Leukocyte Immunoglobulin-Like Receptor subfamily B member 2 (LILRB2), S100 calcium-binding protein A9 (S100A9) and TNF. Of these biomarkers, IL-1Ra, IL-10 and IL-10 mRNA are considered as anti-inflammatory, HLA-DR mRNA, CD74 mRNA, CD3 mRNA and LILRB2 mRNA as markers of adaptive immunity, and peptidoglycan as a pathogen-associated molecular pattern. All other above-mentioned biomarkers are considered as mediators or markers of inflammation.

Plasma (EDTA) was prepared at reception, aliquoted and frozen. Concentrations of plasma markers were determined at the end of the study by sandwich ELISA or by Multiplex analysis according to manufacturers' recommendations (S2 Table). All the plasma and cell surface markers were purchased from providers, except Soluble B7-H6 (S2 Table). All primers and probe for the RNA markers were designed internally and purchased from Eurogentec. However, primers and probe (different designs) can also be bought from some providers like ThermoFisher.

PAXgene® blood samples were incubated at room temperature for 2 hours before freezing. RNA was extracted using the PAXgene® blood RNA kit (PreAnalytix) and frozen at -80°C. Two hundred nanograms RNA were reverse transcribed (RT) using the SuperScript® VILO™ cDNA Synthesis Kit (Life Technologies) at the

end of the study. A RNA calibrator made from PAXgene® samples collected from a pool of healthy volunteers and stimulated ex vivo by LPS was used in each RT run. cDNA was then diluted at 1/20 and stored at -20°C. Polymerase Chain Reaction (PCR) was performed on a LightCycler instrument using the standard Taqman Fast Advanced Master Mix PCR kit according to the manufacturer's instructions (Roche Molecular Biochemicals). Thermocycling was performed in a final volume of 20 µL containing 0.5 µM of primers and 0.1 µM of probe (see primer and probe designs in S4 Table). PCR was performed with an initial denaturation step of 10 min at 95°C, followed by 45 cycles of a touchdown PCR protocol (10 sec at 95°C, 29 sec annealing with 68°C for the first cycle and decrease of 0.5°C for each cycle until reaching 58°C, and 1 sec extension at 72°C). The Second Derivative Maximum Method was used by the LightCycler software to automatically determine the crossing point (cycle threshold, Ct). Ct were converted to Calibrated Normalized Relative Quantity (CNRQ) taking HPRT1 and PPIB as reference genes (1). The distribution of missing values and determination of the cut-off to create binary variables are provided in (2).

Table A: STROBE—Checklist of items that should be included in reports of cohort studies

An Explanation and Elaboration article discusses each checklist item and gives methodological background and published examples of transparent reporting. The STROBE checklist is best used in conjunction with this article (freely available on the Web sites of PLoS Medicine at http://www.plosmedicine.org/, Annals of Internal Medicine at http://www.annals.org/, and Epidemiology at http://www.epidem.com/). Information on the STROBE Initiative is available at http://www.strobe-statement.org.

	Item No	Recommendation	Page No
Title and abstract	1	(*a*) Indicate the study's design with a commonly used term in the title or the abstract	1
		(*b*) Provide in the abstract an informative and balanced summary of what was done and what was found	4
**Introduction**			
Background/rationale	2	Explain the scientific background and rationale for the investigation being reported	5
Objectives	3	State specific objectives, including any prespecified hypotheses	5-6
**Methods**			
Study design	4	Present key elements of study design early in the paper	6
Setting	5	Describe the setting, locations, and relevant dates, including periods of recruitment, exposure, follow-up, and data collection	7
Participants	6	(*a*) Give the eligibility criteria, and the sources and methods of selection of participants. Describe methods of follow-up	7
		(*b*) For matched studies, give matching criteria and number of exposed and unexposed	
Variables	7	Clearly define all outcomes, exposures, predictors, potential confounders, and effect modifiers. Give diagnostic criteria, if applicable	8-9
Data sources/ measurement	8*	For each variable of interest, give sources of data and details of methods of assessment (measurement). Describe comparability of assessment methods if there is more than one group	8
Bias	9	Describe any efforts to address potential sources of bias	8
Study size	10	Explain how the study size was arrived at	6
Quantitative variables	11	Explain how quantitative variables were handled in the analyses. If applicable, describe which groupings were chosen and why	10-11
Statistical methods	12	(*a*) Describe all statistical methods, including those used to control for confounding	
		(*b*) Describe any methods used to examine subgroups and interactions	
		(*c*) Explain how missing data were addressed	10-13
		(*d*) If applicable, explain how loss to follow-up was addressed	
		(*e*) Describe any sensitivity analyses	
**Results**			
Participants	13*	(a) Report numbers of individuals at each stage of study—eg numbers potentially eligible, examined for eligibility, confirmed eligible, included in the study, completing follow-up, and analysed	13
		(b) Give reasons for non-participation at each stage	Fig 1
		(c) Consider use of a flow diagram	
Descriptive data	14*	(a) Give characteristics of study participants (eg demographic, clinical, social) and information on exposures and potential confounders	
		(b) Indicate number of participants with missing data for each variable of interest	(Fig.1)
		(c) Summarise follow-up time (eg, average and total amount)	Fig.1
Outcome data	15*	Report numbers of outcome events or summary measures over time	NA
Main results	16	(*a*) Give unadjusted estimates and, if applicable, confounder-adjusted estimates and their precision (eg, 95% confidence interval). Make clear which confounders were adjusted for and why they were included	16
		(*b*) Report category boundaries when continuous variables were categorized	All tables and fig. 1
		(*c*) If relevant, consider translating estimates of relative risk into absolute risk for a meaningful time period	
Other analyses	17	Report other analyses done—eg analyses of subgroups and interactions, and sensitivity analyses	NA
**Discussion**			
Key results	18	Summarise key results with reference to study objectives	23-26
Limitations	19	Discuss limitations of the study, taking into account sources of potential bias or imprecision. Discuss both direction and magnitude of any potential bias	
Interpretation	20	Give a cautious overall interpretation of results considering objectives, limitations, multiplicity of analyses, results from similar studies, and other relevant evidence	
Generalisability	21	Discuss the generalisability (external validity) of the study results	
**Other information**			
Funding	22	Give the source of funding and the role of the funders for the present study and, if applicable, for the original study on which the present article is based	In the submission system

*Give information separately for exposed and unexposed groups.

Table B: Characteristics of the patients that were discharged or died before day3 (n=33)

PIRO category		Variable	n (%) or med [Q1-Q3]
Predisposition		Age (years)	69.0 [52.0-84.0]
		Male sex	19 (59)
		BMI (kg/m²)	25.7 [21.6-30.0]
		Mc Cabe score, % prediction > 5 years	33 (100)
Insult	At inclusion	Confirmed infection	15 (55)
Response	At inclusion	Temperature (°C)	37.8 [36.3-38.2]
		Lymphocyte count (/mm³)	970 [700-1120]
		Blood platelets (10³/mm³)	155 [100-191]
		Prothrombin time (%)	65 [33-81]
		Blood lactates (meq/L)	2.9 [1.7-10.6]
		PaO2 (mmHg)	119 [79-216]
		FiO2 (%)	70 [45-100]
		PaC02 (mmHg)	42 [34-47]
		Serum creatinin (µmol/L)	126 [67-277]
		Blood hematocrit (%)	35.8 [28.8-41.3]
		White blood cell count (/mm³)	9,900 [3,460-14,760]
		Mean arterial pressure (mmHg)	70 [47-85]
		Urinary output (L/24h)	0.26 [0.00-1.00]
		SAPS II score (points)	55 [50-59]
Organ failure	At inclusion	Total SOFA score (points)	10 [5-14]
		Respiratory SOFA score (points)	2 [1-3]
		Neurological SOFA score (points)	0 [0-4]
		Circulatory SOFA score (points)	3 [0-4]
		Hepatic SOFA score (points)	0 [0-2]
		Coagulation SOFA score (points)	1 [0-1]
		Kidney SOFA score (points)	3 [0-4]
	During the ICU stay	Mechanical ventilation	22 (76)
		Non invasive ventilation	0 (0)
		Vaso-active drugs	22 (76)
		Renal replacement therapy	9 (28)
		Low doses steroid therapy	8 (24)

Table C: Circulating biomarkers with a loading > 0.40 or < -0.40 in each main independent patterns obtained after principal component analysis

Pattern	#1		#2		#3		#4		#5	
Proportion of the variance	30,7%		9,7%		8,0%		5,5%		4,7%	
	Biomarker	*Loading*	*Biomarker*	*Loading*	*Biomarker*	*Loading*	*Biomarker*	*Loading*	*Biomarker*	*Loading*
	IL-6	0.75	IL-10	0.73	LILRB2 RNA	0.79	Galectin-9	0.73	RANTES	0.73
	IL-10 RNA	0.74	IL-1Ra	0.72	TNF-α RNA	0.78	SuPAR	0.62	sTREM-1	-0.47
	S100A9 RNA	0.74	IL-15	0.63	IL-1β RNA	0.77	MIF	0.59		
	HLA-DR RNA	-0.71	IL-8	0.63	CD74 RNA	0.61	Ferritin	0.55		
	GM-CSF	0.70	TNF-α	0.60	CX3CR1 RNA	0.57	IP-10	0.48		
	CD74 RNA	-0.66	MCP-1	0.58	HLA-DR RNA	0.51	MMP-8	0.43		
	CD3D RNA	-0.64	Visfatin	0.57			IL-18	0.41		
	IL-8	0.63	GM-CSF	0.56						
	MCP-1	0.61	MIP-1β	0.54						
	CRP	0.61	sB7-H6	-0.50						
	CX3CR1 RNA	-0.60	IL-18	0.47						
	PSP	0.60	IL-6	0.45						
	PCT	0.57	G-CSF	0.44						
	MMP-8	0.56	IP-10	0.43						
	MIP-1β	0.51								
	IL-1Ra	0.42								

Legend: The 5 patterns where those which explained the largest part of the variance (59 % in total). For each pattern, the individual biomarkers with high loadings are displayed. The individual biomarkers are sorted by the absolute value of their loading. Loadings are interpretable as correlation coefficients between patterns and original biomarkers.

Table D: Value of each pattern of biomarkers in the identified clusters (med [Q1-Q3])

**Patients**	**Cluster 1**		**Cluster 2**		**Cluster 3**		**Cluster 4**		**Cluster 5**		**Cluster 6**		
	n = 86		n = 43		n = 34		n = 18		n = 11		n = 11		
Biomarkers	med	[Q1-Q3]	med	[Q1-Q3]	med	[Q1-Q3]	med	[Q1-Q3]	med	[Q1-Q3]	med	[Q1-Q3]	p value
Pattern 1	-0.73	[-1.04;-0.38]	1.24	[0.8;1.72]	0.14	[-0.45;0.68]	-0.22	[-0.89;0.10]	0.03	[-0.40;0.47]	0.23	[-0.19;0.84]	*0.0001*
Pattern 2	-0.13	[-0.44;0.33]	-0.31	[-0.79;0.23]	-0.45	[-0.84;-0.02]	-0.65	[-1.05;-0.40]	0.32	[-0.35;0.53]	2.54	[1.33;3.31]	*0.0001*
Pattern 3	0.23	[-0.28;0.52]	0.4	[0.09;0.90]	-0.28	[-0.56;0.69]	-1.29	[-1.84;-0.74]	1.1	[0.64;1.82]	-0.41	[-2.32;-0.06]	*0.0001*
Pattern 4	-0.3	[-0.99;0.08]	-0.75	[-1.16;-0.20]	1.05	[0.64;1.45]	0.4	[0.07;1.07]	0.9	[0.49;1.86]	1.11	[0.20;1.40]	*0.0001*
Pattern 5	0.06	[-0.38;0.37]	-0.2	[-0.64;0.32]	-0.73	[-1.09;-0.40]	0.83	[0.47;1.47]	1.76	[0.54;2.86]	-1.03	[-1.72;-0.14]	*0.0001*

Legend: In each raw, the dark red color indicates the highest value and dark blue color indicates the lowest value.

References

1. Hellemans J, Mortier G, De Paepe A, Speleman F, Vandesompele J. qBase relative quantification framework and software for management and automated analysis of real-time quantitative PCR data. *Genome Biol* 2007;8:R19.
2. Parlato M, Philippart F, Rouquette A, Moucadel V, Puchois V, Blein S, Bedos J-P, Diehl J-L, Hamzaoui O, Annane D, Journois D, Ben Boutieb M, Estève L, Fitting C, Treluyer J-M, Pachot A, Adib-Conquy M, Cavaillon J-M, Misset B, Jacqmin S, Lagrange A, de Pinot de Villechenon G, Aissaoui N, Guerot E, Venot M, Prat D, Sztrymf B, Maxime V, Polito A, *et al.* Circulating biomarkers may be unable to detect infection at the early phase of sepsis in ICU patients: the CAPTAIN prospective multicenter cohort study. *Intensive Care Med* 2018;44:1061–1070.
